# Structural insights into DNA N^6^-adenine methylation by the MTA1 complex

**DOI:** 10.1038/s41421-022-00516-w

**Published:** 2023-01-20

**Authors:** Junjun Yan, Feiqing Liu, Zeyuan Guan, Xuhui Yan, Xiaohuan Jin, Qiang Wang, Zican Wang, Junjie Yan, Delin Zhang, Zhu Liu, Shan Wu, Ping Yin

**Affiliations:** 1 National Key Laboratory of Crop Genetic Improvement and National Centre of Plant Gene Research, Hubei Hongshan Laboratory, Huazhong Agricultural University, Wuhan, Hubei China; 2 State Key Laboratory of Biocatalysis and Enzyme Engineering, Hubei Collaborative Innovation Center for Green Transformation of Bio-Resources, Hubei Key Laboratory of Industrial Biotechnology, School of Life Sciences, Hubei University, Wuhan, Hubei China

**Keywords:** Cryoelectron microscopy, DNA methylation

## Abstract

N^6^-methyldeoxyadenine (6mA) has recently been reported as a prevalent DNA modification in eukaryotes. The *Tetrahymena thermophila* MTA1 complex consisting of four subunits, namely MTA1, MTA9, p1, and p2, is the first identified eukaryotic 6mA methyltransferase (MTase) complex. Unlike the prokaryotic 6mA MTases which have been biochemically and structurally characterized, the operation mode of the MTA1 complex remains largely elusive. Here, we report the cryogenic electron microscopy structures of the quaternary MTA1 complex in S-adenosyl methionine (SAM)-bound (2.6 Å) and S-adenosyl homocysteine (SAH)-bound (2.8 Å) states. Using an AI-empowered integrative approach based on AlphaFold prediction and chemical cross-linking mass spectrometry, we further modeled a near-complete structure of the quaternary complex. Coupled with biochemical characterization, we revealed that MTA1 serves as the catalytic core, MTA1, MTA9, and p1 likely accommodate the substrate DNA, and p2 may facilitate the stabilization of MTA1. These results together offer insights into the molecular mechanism underpinning methylation by the MTA1 complex and the potential diversification of MTases for N^6^-adenine methylation.

## Introduction

Chemical modification of DNA and RNA in general, and methylation in particular, plays pivotal roles in regulating various biological processes in organisms across kingdoms^1,2^. The most common and best-studied nucleotide methylations are DNA C5-cytosine methylation (5mC) and RNA N^6^-adenosine methylation (m^6^A), and the writers (methyltransferases, MTases), erasers (demethylases), and readers (methyl-binding proteins) mediating the methyl installation, removal, and recognition, respectively, have been well characterized^3,4^. Apart from 5mC and m^6^A, another prevalent methylation type found in both prokaryotes and eukaryotes is N^6^-methyldeoxyadenine (6mA)^5–9^. Initially discovered to be abundant in prokaryotic genomes, 6mA is exploited as a universal signal for virus defense, cell cycle control, and host–pathogen interaction^10^. The 6mA levels (6mA/A) are reported to be high in some unicellular eukaryotes^7,11–13^, while it remains controversial whether 6mA exists in multicellular eukaryotes^14–23^. The installation of 6mA on DNA is mediated by 6mA MTases. Thus far, several prokaryotic 6mA MTases have been identified and structurally characterized, including M.TaqI, Dam, and CcrM^24–29^. In eukaryotes, a number of 6mA MTases have been predicted based on bioinformatic analysis; however, the MTase activity of these candidates needs further robust biochemical verification^6^.

**Fig. 1:**
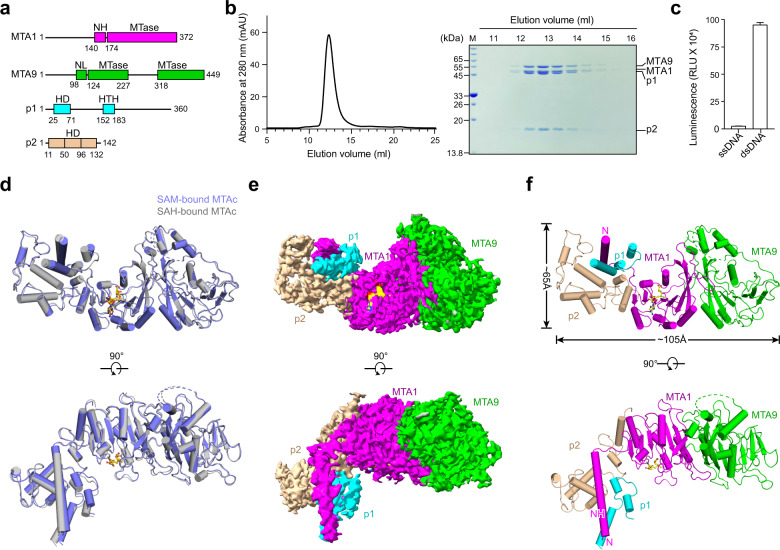
Overall structure of the co-factor substrate-bound MTA1 complex. **a** Schematic diagram of the domain information of the MTA1 complex. NH, N-terminal helix; NL, N-terminal loop; HD, homeobox-like domain; HTH, helix-turn-helix-like domain. **b** Left panel, a representative gel filtration chromatography of the MTA1 complex; right panel, SDS-PAGE gel showing proteins present in the peak fractions of the MTA1–MTA9–p1–p2 heterotetramer from the left panel. **c** SAM-dependent methyltransferase assay (MTase-Glo assay) using 27-bp oligonucleotides. The error bars represent the SEM of three independent measurements. The experiment was repeated twice. ssDNA, single-stranded DNA; dsDNA, double-stranded DNA. **d** Superposition of the SAM-bound (slate) and SAH-bound (gray) MTA1 complexes. SAM and SAH are shown as yellow and orange balls-and-sticks, respectively. **e** Cryo-EM density of the SAM-bound MTA1 complex. MTA1, MTA9, p1, p2, and SAM are shown in magenta, green, cyan, wheat, and yellow, respectively. **f** Atomic model of the MTA1 complex in cartoon, with the same color scheme as **e**.

The first experimentally validated eukaryotic 6mA MTase complex is the MTA1 complex identified in ciliate *Tetrahymena thermophila*. MTA1 complex-mediated 6mA modification directly disfavors nucleosome deposition in vitro, and the disruption of the MTA1 complex severely impacts *T. thermophila* gene expression, cell growth, and sexual development^30,31^. The MTA1 complex comprises four protein subunits, namely MTA1, MTA9, p1, and p2^30^ (Fig. 1a). Both MTA1 and MTA9 contain the MT-A70 domain that is widely involved in eukaryotic RNA m^6^A modification^32^, but only MTA1 contains the conserved catalytic DPPW motif, which is reminiscent of the heterodimeric RNA m^6^A MTase subunits METTL3 and METTL14^33–35^. Neither MTA1 nor MTA9 has a nucleic acid binding domain, and p1 and p2, two homeobox-like DNA-binding proteins, are required for the MTA1 complex’s MTase activity^30^. Interestingly, while the four subunits of the MTA1 complex are conserved among ciliates, green algae, and basal fungi, MTA1 and MTA9 are phylogenetically distinct from all other previously identified m^6^A and 6mA MTases^30,31^. The other two subunits p1 and p2 have no functional homologs in vertebrates^30^. Although the crystal structures of MTA1, MTA1–MTA9 binary complex, and MTA1–p1–p2 ternary complex have just been reported^36^, the overall structure of the quaternary complex is missing. Structural investigations into the whole MTA1 complex will help elucidate the operation mode of the four subunits and contribute to the better understanding of eukaryotic 6mA MTases.

In this study, we determined the cryogenic electron microscopy (cryo-EM) structures of the MTA1 complex in the presence of S-adenosyl methionine (SAM) and S-adenosyl homocysteine (SAH), respectively. In both structures, MTA1, MTA9, p1, and p2 form a heterotetramer with the stoichiometric ratio of 1:1:1:1. The MTA1 and MTA9 MTase domains stack closely against each other, and p1 and p2 interact with a long α-helix in MTA1 away from MTA9. Utilizing an integrative approach based on AlphaFold predictions and structural restraints from intermolecular chemical cross-linking mass spectrometry (CXMS), we further modeled a near-complete structure of the quaternary MTA1 complex. Our structural and biochemical data provide insights into MTase diversification and offers a framework for further characterization of the 6mA MTase complex in eukaryotes.

## Results

### Overall structures of SAM- and SAH-bound MTA1 complexes

We first reconstituted the quaternary MTA1 complex through co-expression of the four subunits MTA1, MTA9, p1, and N-terminally 6×His-tagged p2. After protein extraction and three-step purification (detailed in Materials and methods), a stable quaternary MTA1 complex with homogeneous behavior on size exclusion chromatography (SEC) and double-stranded DNA (dsDNA) methylation activity was obtained (Fig. 1b, c). This quaternary complex was further used for cryo-EM sample preparation and subsequent structural analysis.

After extensive cryo-EM screening (detailed in Materials and methods), we obtained SAM- and SAH-bound MTA1 complexes and determined their structures at average resolutions of 2.6 Å and 2.8 Å, respectively (Supplementary Fig. S1a–c and Table S1). The structures of the two co-factor-bound MTA1 complexes are almost identical with a root-mean-squared deviation (RMSD) of 0.858 Å over 632 Cα atoms, with each structure containing one copy of each of MTA1, MTA9, p1, and p2 (Fig. 1d–f). In both structures, we observed the density for the co-factor substrate (SAM or SAH), MTA1 residues 140–372, MTA9 residues 98–227 and 318–449, the p1 helix-turn-helix-like motif (residues 152–183), p2 residues 11–132 (containing the homeobox-like domain of residues 50–96). Other regions of the four subunits, including the predicted p1 N-terminal homeobox-like domain (residues 25–71) and MTA9 residues 228–317 within the MTase domain, were missing, probably due to flexibility (Fig. 1a, e; Supplementary Fig. S2a–d). As the two structures are almost identical, for simplicity we focused on the higher-resolution SAM-bound MTA1 complex and described the structures of the components in detail.

The SAM-bound MTA1 complex overall exhibits an “L” shape architecture, with MTA1–MTA9 and p1–p2 perpendicular to each other (Fig. 1f). MTA1 is observed to bridge the other three subunits through two distinct regions. Specifically, MTA1 shows extensive contacts with MTA9 on one side, while on the other side the MTA1 N-terminal helix (NH) protrudes from the direction perpendicular to the surfaces of MTA1 and MTA9 and directly interacts with p1 and p2 (Fig. 1f).

### MTA1–MTA9 adopts folds similar to METTL3–METTL14

**Fig. 2:**
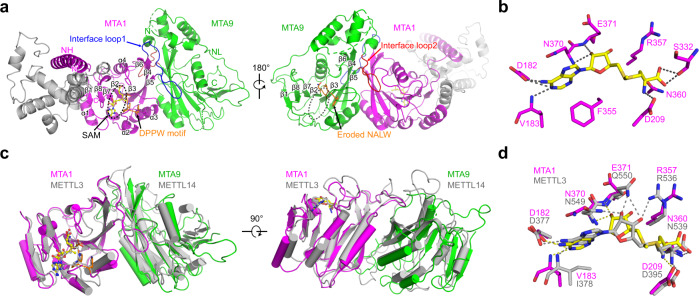
Structural analysis of MTA1–MTA9 in the MTA1 complex. **a** Overall structure of MTA1–MTA9 in the MTA1 complex. MTA1, magenta; MTA9, green; MTA1 interface loop 1, blue; MTA9 interface loop 2, red. The bound SAM is illustrated as a yellow ball-and-stick surrounded by a dashed ellipse. The DPPW motif and the eroded NALW motif are shown as orange sticks. **b** Schematic representation of the interactions between MTA1 and SAM. Residues are shown as magenta sticks. SAM is shown as yellow sticks. Hydrogen bonds are shown as black dashed lines. **c** Superposition of the core MTase domains of MTA1–MTA9 (magenta and green) and METTL3–METTL14 (gray). The bound SAM in MTA1 and METTL3 are shown as yellow and gray balls-and-sticks, respectively. The DPPW motifs in MTA1 and METTL3 are shown as orange and gray sticks, respectively. **d** Superposition of the SAM molecules and the SAM interacting residues in MTA1 and METTL3, with the same color scheme as **c**. Hydrogen bonds in MTA1 and METTL3 are shown as yellow and gray dashed lines, respectively.

In the MTA1 complex, MTA1 and MTA9 directly interact and form a butterfly-like shape (Fig. 2a). A long helix (MTA1 NH; residues 140–173) and an N-terminal loop (MTA9 NL; residues 98–123) are located at the N-terminus of MTase domains of MTA1 and MTA9, respectively (Fig. 1a). The MTA1 NH is away from the two MTase domains, while the MTA9 NL extends across its MTase domain and to the MTase domain of MTA1 (Fig. 2a; Supplementary Fig. S3a). The MTA1 MTase domain adopts a classic α/β sandwich fold comprising a central eight-stranded β-sheet with a strand order of β1↑, β8↑, β7↑, β2↑, β3↑, β5↓, β4↑, and β6↑ surrounded by four α-helices (α1, α2, and α3 on one side, and α4 on the other side) and two 3_10_ helices (Fig. 2a; Supplementary Fig. S3a). The MTA9 MTase domain adopts a fold similar to that of MTA1 excluding its less conserved region (α2, α3, η4) between β4 and β5 (Supplementary Fig. S3a, b).

The antiparallelly arranged MTA1 and MTA9 C-terminal MTase domains stack closely against each other through extensive hydrogen bonds and hydrophobic interactions, with a buried surface area of ~1877 Å^2^ (Supplementary Fig. S3a). These interactions are primarily mediated by two interface loops from MTA1 and MTA9, respectively (Fig. 2a; Supplementary Fig. S4a). The interface loop 1 in MTA1 (residues 282–299) mainly interacts with MTA9 β4 and the loop between MTA9 β5 and β6 (Supplementary Fig. S4b). Similarly, the interface loop 2 in MTA9 (residues 348–359) interacts with MTA1 β4 and the loop between MTA1 β5 and β6 (Supplementary Fig. S4c).

The co-factor substrate SAM is found in the MTase domain of MTA1. Specifically, the SAM molecule is positioned at the end of MTA1 β2, β7, and β8, facing the conserved DPPW catalytic motif (residues 209–212) in MTA1 (Fig. 2a). This is coordinated by the surrounding MTA1 residues via extensive hydrogen bonds. The adenine moiety of SAM is recognized by the side chain of D182 and the main chain of V183 in MTA1. The hydroxyl groups of ribose are surrounded by MTA1 N370, E371, and R357. Several residues, including D209, S332, and N360, directly contact the methionine moiety of SAM (Fig. 2b). In MTA9, SAM is not found and an eroded NALW motif is observed instead of the conventional catalytic DPPW motif (Fig. 2a; Supplementary Fig. S5a). The MTA1 D209A mutation (in the catalytic DPPW motif) almost abolished the MTase activity of the complex, and the MTA9 N150A mutation (in the eroded NALW motif) showed no obvious impact on the MTase activity (Supplementary Fig. S5b). These findings corroborate the previous hypothesis that MTA1 is the catalytic center and mediates the transfer of the methyl group from SAM to the acceptor adenine moiety^30^.

The METTL3–METTL14 RNA m^6^A MTase core complex consists of one active MTase domain and the other is inactive and MTase-like. The SAM molecule was found in METTL3 but not in METTL14 in the solved structures^33–35^. Biochemical characterization further demonstrated that METTL3 is the catalytic subunit and METTL14 facilitates the binding of substrate RNA^33–35^. MTA1–MTA9 and METTL3–METTL14 showed overall sequence similarity and superposition of their MTase domains revealed a RMSD of ~3.0 Å over 352 Cα atoms (Fig. 2c; Supplementary Fig. S5a). Moreover, the loops (containing the DPPW motif) that surround the SAM-binding pocket in MTA1 as well as the surrounding residues for SAM coordination display conformations almost identical to those in METTL3 (Fig. 2c, d). All the characteristics of the MTA1–MTA9 MTase domains shows high similarity to those of METTL3–METTL14, supporting the view that they belong to the same class of MTase^37^.

### MTA1 NH contributes to the assembly of MTA1–MTA9 with p1 and p2

**Fig. 3:**
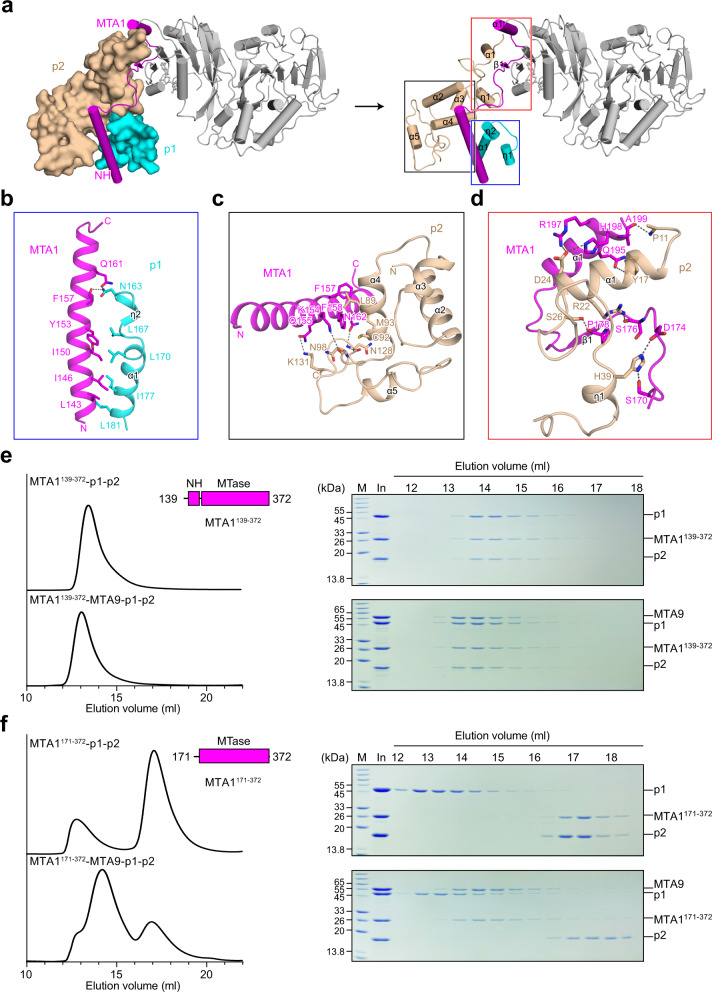
The MTA1 NH contributes to the assembly of MTA1–MTA9 with p1 and p2. **a** Structure of the NH-bound p1 and p2 in the MTA1 complex. Three interaction interfaces (shown in blue, black, and red rectangles, respectively) can be observed between MTA1 (magenta), p1 (cyan), and p2 (wheat). **b**–**d** Close-up views of the insets shown in **a**. **e**, **f** SEC analysis for the truncated versions of MTA1. MTA1^139–372^ containing the NH can interact with p1 and p2, further forming the quaternary MTA1^139–372^–MTA9–p1–p2 complex with MTA9 (**e**). MTA1^171–372^ without the NH retained the binding capacity with MTA9, but showed no interaction with p1 and p2 (**f**).

The two potential DNA-binding proteins p1 and p2 interact with MTA1 in the complex, of which the interaction interfaces are opposite to that of MTA1–MTA9 (Fig. 3a). The p1 protein residues 152–183 form a helix-turn-helix-like motif consisting of 3_10_ helices η1 and η2 and α-helix α1 (Fig. 3a). The p2 protein exhibits an extended conformation comprising the N-terminal α-helix α1 and 3_10_ helix η1, a central homeobox-like domain (including α2, α3, and α4), and the C-terminal α-helix α5 with surrounding loops (Fig. 3a). The NH of MTA1 interacts with p1 and p2, respectively (Fig. 3a). Specifically, for MTA1 and p1, the N-terminal region of the MTA1 NH intertwines with the p1 α1 mainly through hydrophobic interactions (Fig. 3b). For MTA1 and p2, the C-terminal region of the MTA1 NH anchors between the p2 homeobox-like domain and the p2 C-terminal region via multiple hydrogen bonds and hydrophobic interactions (Fig. 3c). The MTA1 α1 and β1 in the MTase domain and the long loop connecting NH and β1 also interact with the N-terminus of p2 through hydrogen bonds (Fig. 3d).

To corroborate the importance of MTA1 NH in driving the complex assembly, truncated versions of MTA1 proteins were generated and subjected to subsequent co-incubation and SEC assays with the other subunits. The N-terminally truncated MTA1 containing the NH (MTA1^139–372^, residues 139–372) can interact with p1 and p2 and form a quaternary complex with MTA9 (Fig. 3e). In contrast, the NH-deleted MTA1 (MTA1^171–372^, residues 171–372) showed no interaction with p1 and p2 while retaining the binding capacity with MTA9 (Fig. 3f). Besides, the N-terminally GST-tagged NH alone showed no interaction with p1 and p2 (Supplementary Fig. S6). These results together suggest that the MTA1 NH contributes to the assembly of MTA1–MTA9 with p1 and p2 and is indispensable for the integrity of the MTA1 complex.

Considering that the MTA1–MTA9 and METTL3–METTL14 MTase domains exhibited high structural similarity, we wondered whether METTL3–METTL14 can form a complex with p1 and/or p2. Neither the full-length METTL3–METTL14 complex nor its MTase domains showed interactions with p1 and p2 (Supplementary Fig. S6). We further generated a chimeric protein MTA1^139–173^–METTL3^369–580^ by fusing the MTA1 NH (residues 139–173) to the N-terminus of the METTL3 MTase domain (residues 369–580) and investigated its interaction with p1 and p2. Interestingly, MTA1^139–173^–METTL3^369–580^ formed a ternary complex with p1 and p2, and further formed a well-behaved quaternary complex with the METTL14 MTase domain (residues 107–395) in a manner similar to the quaternary MTA1 complex (Supplementary Fig. S6). These findings re-emphasized the important role that the MTA1 NH plays in the assembly of the MTA1 complex and the underlying relationships between the MTA1 complex and the METTL3–METTL4 complex.

### AI-empowered integrative structural characterization of the MTA1 complex

**Fig. 4:**
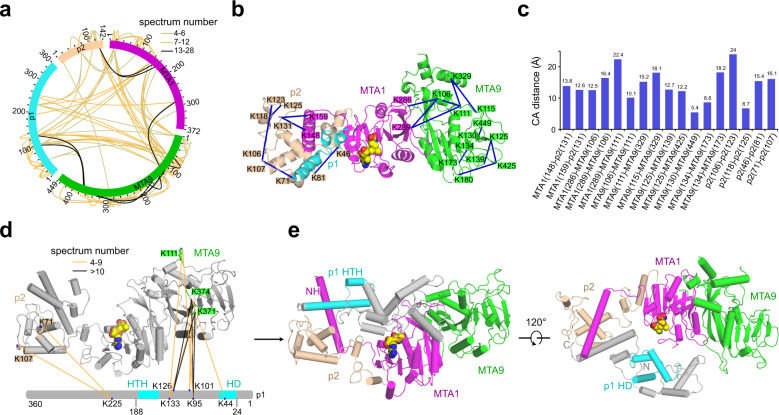
Integrative modeling of a near-complete MTA1 complex based on CXMS data and AlphaFold prediction. **a** The 108 non-redundant cross-links identified in the MTA1 complex shown in a circle plot. The numbers of the corresponding spectra of each cross-link were indicated by the colors of the line. Cross-links were filtered with the following criteria: FDR < 0.05 at the spectrum level, supervised vector machine (SVM) value < 1 × 10^–2^ and spectral counts ≥ 4. **b** Cross-links mapped on the structure of the MTA1 complex. Residues in MTA1, MTA9, and p2 involved in the cross-linked pairs are shown in magenta, green, and wheat rectangles, respectively. SAM is illustrated as a yellow space-filling representation. **c** The distance distribution of all cross-links mapped to the structure of the MTA1 complex. The 26 Å cutoff value was used to filter the cross-links, well below the 35-Å upper limit. **d** The inter-subunit cross-links between MTA1–MTA9–p2 and p1 in the MTA1 complex. HD, homeobox-like domain; HTH, helix-turn-helix-like motif. **e** The modeled near-complete MTA1 complex. MTA1, MTA9, p1, and p2 are shown in magenta, green, gray, and wheat, respectively. The p1 HD (residues 25–71) and HTH (residues 152–183) are highlighted in cyan in the two different views, respectively. Residues 24–188 of p1 was docked to MTA1–MTA9–p2 in the MTA1 complex.

The homeobox-like proteins p1 and p2 have been suggested to mediate the DNA binding of the MTA1 complex^30^. As mentioned above, in the solved quaternary complex structures, we observed most residues of p2 yet a major region of p1 (including the predicted p1 N-terminal homeobox-like domain) was missing. Thus, to gain more insights into the DNA binding and methylation by the complete MTA1 complex, we further conducted integrative modeling based on the solved structures, the data from in vitro CXMS of the intact complex, and AI-based predictions. Specifically, for the in vitro CXMS, we identified a total of 108 cross-links (53 intra-subunit cross-links and 55 inter-subunit cross-links) in the MTA1 complex with the false discovery rate (FDR) < 5% and spectrum number ≥ 4 (Fig. 4a). Among the identified cross-links, 17 could be directly compared with matching residues in the model, whereas the other cross-links were on flexible regions without electron density and not modeled. The structural information indicated by the 17 cross-links is consistent with the resolved structure of the MTA1 complex (Cα–Cα distances < 26 Å; Fig. 4b, c), indicating that the obtained CXMS data is reliable.

A total of 12 cross-links between p1 and the other subunits (two cross-links between p1 and p2, and 10 cross-links between p1 and MTA9) were identified in the MTA1 complex (Fig. 4d). Notably, for p1 and MTA9, the residues close to the N-terminus of p1 (including the homeobox-like domain residues 25–71) were cross-linked with the MTase domain of MTA9. These MTA9–p1 cross-linked pairs include K111–K133 (K111 from MTA9 and K133 from p1), K111–K95, K371–K101, K371–K126, K371–K133, K371–K95, K374–K101, K374–K126, K374–K44, and K374–K95. One residue close to the C-terminus of p1 was cross-linked with the C-terminal region of p2 (including p1–p2 cross-linked pairs K225–K71 and K225–K107; Fig. 4d). The intra-subunit cross-links in p1 are all located around its N-terminal homeobox-like domain, which is consistent with the AlphaFold prediction that p1 contains an N-terminal structured region (Supplementary Fig. S7a). Moreover, the AlphaFold-predicted structure of the helix-turn-helix-like motif in p1 (residues 152–183) and the solved structure are almost identical (Supplementary Fig. S7b). Therefore, we docked the AlphaFold-predicted structure of p1 (24–188), which comprises the N-terminal structured region, the central flexible loop, and the C-terminal helix-turn-helix-like motif, with MTA1–MTA9–p2 in the solved MTA1 complex structure based on the CXMS information (Fig. 4d and Supplementary Fig. S7a; detailed in Materials and methods). In the highest-ranking model, the p1 helix-turn-helix-like motif was docked in close proximity to the MTA1 NH, which is in agreement with the structure of the MTA1 complex (Fig. 4e). The p1 N-terminal structured region was docked close to the MTase domains of MTA1 and MTA9, with the homeobox-like domain facing the MTA1–MTA9 interaction interface (Fig. 4e).

### Analysis of the potential DNA-binding surfaces in the MTA1 complex

Utilizing the highest-ranking near-complete MTA1 complex structural model as a reference, we investigated the potential DNA-binding surfaces in the MTA1 complex through bioinformatical, structural, and biochemical analyses. We first examined the p1–p2 in the complex, which is suggested to bind DNA by previous studies^30,36^. The p2 homeobox-like domain (residues 50–96) resembles the homeodomain of the yeast α2 repressor MATα2 (PDB: 1APL) with three α-helices packing against one another to form a compact conformation (Supplementary Fig. S8a, b). In the structure of the MATα2 homeodomain–DNA complex, several positively charged residues in the third helix α3 contact with the bases and with the sugar-phosphate backbone of the DNA^38^. However, in the p2 homeobox-like domain, the corresponding helix α4 is characterized by a negatively charged surface and interacts with the MTA1 NH in the MTA1 complex (Fig. 3c; Supplementary Fig. S8a–c). Thus, p2 interacts with MTA1 and likely stabilizes it for SAM binding^36^. In contrast, the surrounding of the α-helix (α3) that may be used for DNA binding in the predicted p1 N-terminal homeobox-like domain (displaying fold similar to that of p2) is rich in positively charged amino acids (Supplementary Fig. S8d, e), and we speculated that p1 may play a role in the substrate DNA binding by the MTA1 complex.

**Fig. 5:**
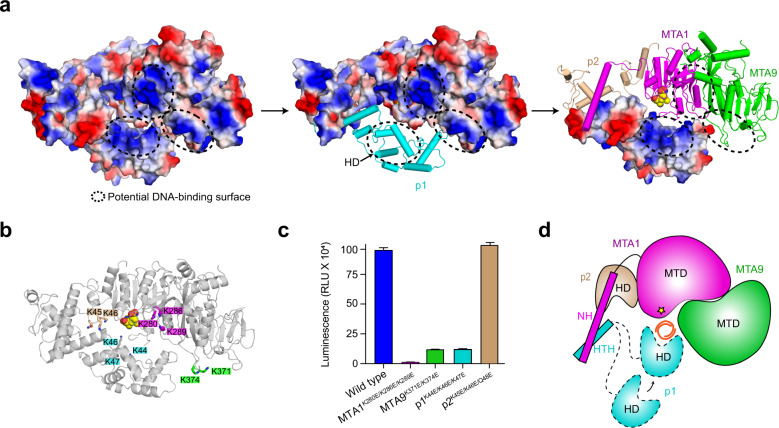
Potential DNA-binding surfaces in the MTA1 complex. **a** The surface electrostatic potential of the MTA1 complex calculated by PyMOL. Three potential DNA-binding surfaces between MTA1 (magenta), MTA9 (green), and p1 (cyan) are highlighted by black dashed ellipses. SAM is illustrated as a yellow space-filling representation. HD, homeobox-like domain. **b** The positively charged residues in the potential DNA-binding surfaces of MTA1, MTA9, p1, and p2 are shown in magenta, green, cyan, and wheat rectangles, respectively. **c** The MTase activities of the wild-type MTA1 complex and the mutant complexes with mutations in the putative DNA-binding surfaces. The indicated mutations were introduced to each of the four subunits: MTA1, K280E/K286E/K289E; MTA9, K371E/K374E; p1, K44E/K46E/K47E; p2, K45E/K46E/Q48E. The error bars indicate the SEM of three independent measurements. The experiment was repeated twice. **d** The proposed working model for DNA 6mA modification by the MTA1 complex. MTA1 (magenta) primarily functions as a catalytic core. MTA9 (green) and p1 (cyan) facilitate DNA binding and p2 (wheat) help stabilize the MTA1. The DNA substrate (orange ribbon) is coordinated by MTA1, MTA9, and p1. The p1 HD and HTH are connected by a flexible loop. SAM is indicated as a yellow star. NH, N-terminal helix; HTH, helix-turn-helix-like motif; MTD, MTase domain.

The surface electrostatic potential of the p1-docked model shows a continuous positively charged surface between MTA1, MTA9, and p1 adjacent to SAM (Fig. 5a). At least eight positively charged residues are found on this surface (K280, K286, and K289 from MTA1, K371 and K374 from MTA9, and K44, K46, K47 from p1; Fig. 5b), and this positively charged surface might be involved in DNA binding. To test this hypothesis, charge-reversal mutational analyses were conducted regarding these MTA1, MTA9, and p1 positively charged residues. As expected, MTase activity was significantly reduced in the mutant complex containing these charge-reversal mutations compared to the wild-type complex (Fig. 5c). The mutations in MTA9 weakened the DNA binding ability of the complex; individual MTA1 or p1 mutations showed no obvious impact while simultaneous mutations in the two subunits significantly reduced the complex’s DNA binding ability (Supplementary Fig. S9). We also introduced mutations to the p2 positively charged residues located at the groove between MTA1, p1, and p2. No obvious change in MTase activity or DNA binding ability was observed for the complex containing the p2 mutant compared to the wild-type complex (Fig. 5c; Supplementary Fig. S9). These results together corroborate the important role of the positively charged surface formed by MTA1, MTA9, and p1 in substrate DNA binding and thus DNA methylation.

## Discussion

In this study, we determined the SAM- and SAH-bound MTA1 complex structures. Our structural and biochemical data revealed that MTA1 is the catalytic core that accommodates SAM/SAH; MTA9 and p1 may facilitate DNA binding of the MTA1 complex. Consistent with the newly reported crystal structures of MTA1 and MTA1–p2^36^, our results also suggest that p2 stabilizes the MTA1 and assists it in binding the methyl donor SAM. Despite that substrate DNA and full-length p1 were used for cryo-EM sample preparation, only the helix-turn-helix-like motif was observed for p1. The p1 positively charged N-terminal region that contains the homeobox-like domain might be involved in DNA binding and is connected to the helix-turn-helix-like motif through a long flexible loop, which may explain why in the solved structure this region and substrate DNA are missing.

Previous studies have implied the dimeric feature of the class-β MTase structures^37^. For instance, two prokaryotic class-β MTases are suggested to form homodimers for their activity^27,29^ and the mammalian m^6^A MTase core complex METTL3–METTL14 function as a heterodimer^33–35^. Here, based on AI-empowered integrative prediction and biochemical characterization, we propose a model of the MTA1 complex for DNA methylation (Fig. 5d). This model is similar to that of the prokaryotic class-β 6mA MTase CcrM (PDB: 6PBD), in which the dimeric CcrM is suggested to employ a “division of labor” between its two subunits^29^. Specifically, as revealed by the DNA-bound CcrM structure, the catalytic domain from one monomer and the target recognition domain (TRD) from the other monomer are face to face and establish a functional surface for DNA recognition and methylation^29^ (Supplementary Fig. S10a, b). Similarly, in the MTA1 complex, MTA1 is the MTase for catalysis and its NH firmly binds the potential DNA-binding protein p2, forming a monomer-like 6mA MTase (MTA1–p2). The MTA1 NH also binds the C-terminal helix-turn-helix like motif of the other potential DNA-binding protein p1. The predicted p1 N-terminal homeobox-like domain is close to MTA9, the other MTase in the MTA1 complex without catalytic activity, potentially forming the other monomer-like 6mA MTase (MTA9–p1). The catalytic domain of MTA1–p2 and the TRD of MTA9–p1 face each other, forming a surface appropriate for the binding of one DNA molecule (Fig. 5d).

Our data indicated that the MTA1 complex from a unicellular eukaryote exhibited overall structural similarity to the METTL3–METTL14 complex^37^. Interestingly, METTL3–METTL14 shows 6mA MTase activity in vitro on single-stranded DNA (ssDNA) and lesion-containing dsDNA^39,40^. In addition, the MTA1 NH-fused METTL3 MTase domain can interact with p1 and p2 and further form a quaternary complex with METTL14 MTase domain (Supplementary Fig. S6). These together support the previously proposed hypothesis that the MTases in the MTA1 complex and the METTL3–METTL14 complex originated from an ancestral class-β MTase and may have undergone evolutionary divergence^37^. The MTases from these complexes harbor the relatively conserved MTase domains, whereas their TRDs (possibly additional regulatory subunits) show diversification. These diversified TRDs may determine the complexes’ binding specificity to their substrate nucleic acids. Notably, METTL3–METTL14 is the catalytic core of a larger m^6^A writer complex which contains additional regulatory subunits including WTAP, VIRMA, and ZC3H13^41^. METTL3–METTL14 complex alone has MTase activity and the m^6^A writer regulatory complex enhances its MTase activity^42,43^. In contrast, all four components of the MTA1 complex are required for MTase activity^30,36^. Our solved p1 and p2 structures in the MTA1 complex are quite different from the newly reported structures of the m^6^A writer regulatory complexes^42,43^ (Supplementary Fig. S10c, d). These further suggest the diversification of eukaryotic MTase complex. We anticipate that the future structural determinations of these two complexes with their substrate nucleic acids will help us fully understand the molecular determinants underlying N^6^-adenine methylation on DNA and RNA in eukaryotes.

## Materials and methods

### Molecular cloning, protein expression, and purification

The codon-optimized complementary full-length cDNAs for subunits of the *Tetrahymena thermophila* MTA1 complex were synthesized by General Biosystems Company. Full-length METTL3 and METTL14 were amplified from the *Homo sapiens* cDNA library. Gibson Assembly method was used for cloning construction. The truncated genes were subcloned using the standard polymerase chain reaction (PCR) method. Site-specific mutagenesis within the subunits of the MTA1 complex and the fusing NH to METTL3 were carried out using the overlap PCR. All the constructs were verified by DNA sequencing.

For the cryo-EM sample preparation and 6mA methylation assay, the full-length cDNAs of MTA1, p1 and p2 were subcloned into the plasmid A using pQlink vector^44^, and the full-length cDNA of MTA9 was subcloned into the plasmid B using pBB75 vector. For mutagenesis analysis, the mutant MTA1 complex was also subcloned into the plasmids A and B vectors, respectively. p2 contains an N-terminal 6×His-tag. The plasmid A with ampicillin resistance and the plasmid B with kanamycin resistance were co-transformed into the BL21 (DE3) strain of *E. coli* for the co-expression of the MTA1 complex. The cells were induced with 0.2 mM isopropyl-β-D-thiogalactoside (IPTG) at 16 °C for 16 h. Harvested cells were lysed by a high-pressure cell disrupter in a buffer containing 25 mM Tris-HCl, pH 8.0, 300 mM NaCl, 5 mM imidazole, and 1 mM phenylmethanesulfonyl fluoride (PMSF). After centrifugation at 14,000 rpm at 4 °C, the supernatant was incubated with Ni^2+^ affinity resin, and the bound protein was eluted with the buffer containing 25 mM Tris-HCl, pH 8.0, 300 mM NaCl, and 400 mM imidazole. The eluted protein was applied to a Heparin HP column (GE Healthcare), followed by a gradient NaCl elution (up to 1.5 M) in 25 mM Tris-HCl (pH 8.0). Target proteins were further purified to homogeneity by gel filtration chromatography (Superdex-200 Increase 10/300 GL column, GE Healthcare) in a buffer containing 25 mM Tris-HCl, pH 8.0, 300 mM NaCl, 5 mM dithiothreitol (DTT) and 1 mM MgCl_2_.

For the MTA1 truncation assay, MTA1 and its truncated forms, as well as p1 and p2 were subcloned into a pET15D vector (Novagen) with an N-terminal 6×His-tag, respectively. MTA9 was subcloned into a pET21b vector (Novagen) with a C-terminal 6×His-tag. Proteins were expressed in *E. coli* strain BL21 (DE3), and induced with 0.2 mM IPTG at 16 °C for 14 h, respectively. Harvested cells were lysed, and the target proteins were purified over Ni^2+^ affinity resin, Heparin HP, and Superdex-200 Increase 10/300 GL columns used in tandem. The protein was finally prepared in a buffer containing 25 mM Tris-HCl, pH 8.0, 300 mM NaCl and 5 mM DTT.

For the NH fusing assay, METTL3, METTL3^369–580^ and MTA1^139–173^–METTL3^369–580^ were subcloned into pET21b vector (Novagen) with a C-terminal 6×His-tag. METTL14, METTL14^107–395^, p1 and p2 were subcloned into pET15D vector with an N-terminal 6×His-tag. MTA1^139–173^ was subcloned into pQlink vector with an N-terminal GST-tag. Proteins were expressed in *E. coli* strain BL21 (DE3), and induced with 0.2 mM IPTG at 16 °C for 14 h, respectively. Cells were collected and homogenized in a buffer containing 25 mM Tris-HCl, pH 8.0, 300 mM NaCl and 1 mM PMSF. The target proteins were purified over Ni^2+^ affinity resin, Heparin HP, and Superdex-200 Increase 10/300 GL columns used in tandem. The protein was finally prepared in a buffer containing 25 mM Tris-HCl, pH 8.0, 300 mM NaCl, and 5 mM DTT.

### DNA sample preparation and 6mA methylation assay

A 27-nt ssDNA (5′-AACTTCTGTCATTACATTAAGCTTTAA-3′, as negative control), and the corresponding dsDNA were used in the 6mA methylation assay. The ssDNA oligonucleotides were synthesized and dissolved in the buffer containing 25 mM Tris-HCl, pH 8.0, and 150 mM NaCl. The ssDNA was mixed with a complementary strand at equal molar amounts, and incubated in boiled water and gradually cooled to room temperature for dsDNA formation.

Reactions were carried out in duplicate with a 15 µL reaction mixture containing 2 μM MTA1 complex, 3.3 μM DNA, 6.6 µM SAM, 33 mM Tris-HCl, pH 8.0, 150 mM NaCl, 5 mM DTT, and 6 mM EDTA at 37 °C for 5 h. Trifluoroacetic acid (TFA) was added at a final concentration of 0.1% (v/v) to terminate the reaction, and an 8-µL mixture was transferred to a Half-Area 384-well plate. An MTase-Glo^TM^ Methyltransferase Assay kit was used to measure activity, which involves converting the reaction by-product SAH into ATP, and detecting ATP through luciferase^45^. The luminescence signal was measured by a TECAN infinite M200 (TECAN).

### Cryo-EM sample preparation and data acquisition

For the SAM-bound MTA1 complex, the purified MTA1 complex at ~4 μM (OD_280_ of ~0.5) was mixed with SAM at the molar ratio of 1:5 and incubated on ice for 1 h. For the SAH-bound MTA1 complex, the purified MTA1 complex was diluted to the final concentration of ~0.25 mg/mL and incubated with the 27-bp dsDNA and SAH at the molar ratio of 1:2:5 and incubated on ice for 1 h. Without any further purification, the above mixtures were prepared directly for grid preparation.

After extensive cryo-EM screening, detergent octyl glucoside (0.025%) was added to the sample immediately before grid freezing to overcome a strong preferred orientation of particles on the grids. Then 3.5 µL sample were deposited onto a freshly glow-discharged holey carbon grid (Quantifoil R1.2/1.3, Cu 300 mesh) and plunged into liquid ethane using an FEI Virobot Mark IV after blotting for 3.5 s with blot force 0, Whatman 597 filter paper at 4 °C and 100% humidity. Each grid was screened using a Thermo Scientific™ Glacios™ Cryo-EM at 200 keV. Cryo-EM data were collected on a Titan Krios TEM operated at 300 keV and equipped with a K3 Summit direct detector (Gatan) positioned to post a GIF quantum energy filter (slit width 20 eV). Automated data acquisition was carried out using EPU in super-resolution mode at a magnified pixel size of 0.85 Å, with defocus values ranging from −1.0 mm to −1.5 mm. The total exposure time was set to 2.51 s with 40 frames, resulting in an accumulated dose of ~55.1 e^–^/Å^2^.

### Cryo-EM data processing, model building, and refinement

The scheme of the data processing pipeline is shown in Supplementary Fig. S1a. For the SAM-bound MTA1 complex, 973,331 particles from 1122 micrographs were automatically picked using the cryoSPARC blob picker^46^. After two-dimensional classification, a total of 945,684 good particles were selected and subjected to several cycles of three-dimensional classification in cryoSPARC^46^. 628,049 particles belonging to the best class were selected, followed by non-uniform refinement and local refinement. The SAM-bound MTA1 complex yielded a cryo-EM density with an estimated resolution of 2.6 Å based on gold standard Fourier shell correlation^47^ (Supplementary Fig. S1b, c).

The dataset of the SAH-bound MTA1 complex was processed by a similar procedure as above. Briefly, 3,536,396 particles were autopicked from 3990 micrographs and 3,382,551 particles were selected for 3D classification after two-dimensional classification. 614,753 particles from the best class were subject to 3D refinement (Supplementary Fig. S1a). The final reconstruction yielded the structure of the complex with an overall resolution of 2.8 Å (Supplementary Fig. S1b, c).

The atomic models for the SAM- and SAH-bound MTA1 complexes were built in COOT^48^ and refined with PHENIX^49^. The two structures were validated through the examination of Molprobity^50^ scores and the Ramachandran plots (Supplementary Table S1).

### SEC assay

The purified proteins for MTA1 truncation assay and NH fusing assay were all mixed in an equimolar ratio and incubated on ice for 50 min. Protein mixtures were then run through a Superdex-200 Increase 10/300 GL column (GE Healthcare) at a flow rate of 0.55 mL/min with the buffer containing 25 mM Tris-HCl, pH 8.0, 300 mM NaCl, and 5 mM DTT. Samples from relevant fractions were analyzed on a 15% SDS-PAGE gel and were visualized with Coomassie blue staining.

### In vitro cross-linking

For cross-linking experiments, the MTA1 complex was purified on a Superdex-200 Increase 10/300 GL column equilibrated with the buffer containing 25 mM HEPES, pH 7.5, 300 mM NaCl, and 5 mM DTT. The cross-linking agent bis(sulfosuccinimidyl) suberate (BS^3^, from Thermo Fisher) was prepared at a concentration of 100 mM in DMSO. BS^3^ was added to the MTA1 complex (~4 µM) at a molar ratio of 100:1. The reaction was performed at room temperature for 30 min and then quenched by the addition of 20 mM Tris-HCl (pH 7.5). The experiments were triplicated with three parallel samples.

### Mass spectrometry and data analysis

The cross-linked protein sample was precipitated with 6 volumes of pre-chilled acetone at 4 °C and centrifuged for 20 min to remove supernatant. The precipitated sample was dissolved in 8 M urea and 0.1 M Tris-HCl (pH 8.5), reduced with 5 mM DTT at 25 °C for 10 min, and alkylated with 10 mM iodoacetamide in dark for 15 min. Subsequently, 3 volumes of Tris-HCl (pH 8.5) were added to dilute the sample, which also contained 1 mM CaCl_2_ (to repress Chymotrypsin activity) and 20 mM methylamine (to reduce the modification of the Carbamate modification at the N-terminus of the peptide segment). Trypsin digestion was carried out at 37 °C overnight with sequencing grade modified trypsin (Promega, mass ratio = 1:20). The reaction was quenched with TFA to a final concentration of 5%.

Trypsin-digested peptides were purified with C18 Spin Tips (Thermo Fisher Scientific) and were analyzed in the Q Exactive HF Hybrid Quadrupole-Orbitrap mass spectrometer (Thermo Fisher Scientific) coupled to an EASY-nLC 1200 liquid chromatography system, with a 75 μm, 15 cm Acclaim PepMap^TM^ RSLC column. The peptides were eluted over a 75 min linear gradient from 95% buffer A (water with 0.1% Formic acid) to 35% buffer B (acetonitrile with 0.1% Formic acid) with a flow rate of 300 nL/min. Each full MS scan (Resolution = 120,000) was followed by 15 data-dependent MS2 (Resolution = 15,000), with a stepped normalized collision energy of 10, 25, and 30. The isolation window was set to 1.6 m/z. Precursors of charge states 3–6 were collected for MS2 scans. Dynamic exclusion window was set to 40 s.

The cross-linking data were analyzed by pLink2^51,52^. The following search parameters were used: MS1 accuracy = ±20 ppm; MS2 accuracy = ±20 ppm; enzyme = trypsin (with full tryptic specificity but allowing up to three missed cleavages); crosslinker = BS^3^ (with an assumed reaction specificity for lysine and protein N-termini); fixed modifications = carbamidomethylation on cysteine; variable modifications = oxidation on methionine, hydrolyzed/aminolyzed BS^3^ from reaction with ammonia or water on a free cross-linker end. The identified candidates have filtered these parameters: FDR < 5%, SVM score < 10^−2^, identified in at least two biological repeat experiments and with total peptide-spectrum matches (PSMs) ≥ 4. The experimental cross-links were illustrated with Crosslink-viewer^53^. Visualization of the cross-links on the structures used PyMOL with the PyXlinkViewer plug-in^54^.

### AI-based structure prediction and CXMS-guided protein–protein docking

The structure model of p1 was predicted using the AlphaFold2 implementation in the ColabFold notebooks running on Google Colaboratory with the default settings^55,56^. CXMS-guided protein–protein docking was carried out using the Expert interface of the HADDOCK server (version 2.4)^57^. The obtained structure of the MTA1–MTA9–p2 heterotrimer in the MTA1 complex and the AlphaFold structure of p1^24–188^ were set as input models for molecular docking. Residues L167, L170, I177, and L181 of p1, and residues L143, I146, I150, and Y153 of MTA1 were set as active residues directly involved in the interaction between the two models according to the structure of the MTA1 complex (Fig. 3b). Distance restraints with ranges of 0–35 Å (Cα–Cα)^58^ were applied to the MTA9–p1 cross-linked pairs K111–K95, K111–K133, K371–K95, K371–K101, K371–K126, K371–K133, K374–K44, K374–K95, K371–K101, and K371–K126. Center-of-mass restraints was enabled and other parameters were set as default in HADDOCK.

### Electrophoretic mobility shift assay (EMSA)

For EMSA, the 27-nt ssDNA oligonucleotides (5′-AACTTCTGTCATTACATTAAGCTTTA A-3′) labeled at the 5′ end with FAM were synthesized by General Biosystems (Anhui) Co. Ltd. and annealed with an equimolar amount of the complementary strand as described above. An aliquot of 500 nM FAM-labeled dsDNA was mixed with increasing concentrations of wild-type or mutant complexes (0–15 μM) in 20 μL buffer containing 25 mM Tris-HCl, pH 7.5, 150 mM NaCl, 20 mM DTT, 0.2 mg/mL BSA, 2 μg/mL Heparin, 50% glycerol and incubated on ice for 20 min. The obtained products were then resolved on 8% native acrylamide gels (37.5:1 acrylamide:bis-acrylamide) in 0.5× Tris-glycine buffer under an electric field of 10 V/cm for 2 h. Gels were visualized using Amersham Typhoon.

## Supplementary information

Supplementary information

## Data Availability

Atomic coordinates and EM densities have been deposited in the Protein Data Bank (PDB) and the Electron Microscopy Data Bank (EMDB) for the reported structures of the SAM-bound MTA1 complex (PDB ID: 7YI9; EMDB ID: EMD-33854) and the SAH-bound MTA1 complex (PDB ID: 7YI8; EMDB ID: EMD-33853). The CXMS data have been deposited to the ProteomeXchange Consortium PRIDE: IPX0005101000.
