# Supplementary material for: Structural insights into DNA N6-adenine methylation by the MTA1 complex
Source: Cell Discov. 2023 Jan 20;9:8. doi: 10.1038/s41421-022-00516-w (PMC9852454; doi:10.1038/s41421-022-00516-w)
Supplement: Supplementary file 1 — Supplementary information [file 41421_2022_516_MOESM1_ESM.pdf]

Supplementary Information for

Structural insights into DNA N^6-adenine methylation by the MTA1 complex

Junjun Yan[1,3], Feiqing Liu[1,3], Zeyuan Guan[1], Xuhui Yan[1], Xiaohuan Jin[1], Qiang Wang[1], Zican Wang[1], Junjie Yan[1], Delin Zhang[1], Zhu Liu[1], Shan Wu[2,*], and Ping Yin[1,*]

[1]National Key Laboratory of Crop Genetic Improvement and National Centre of Plant Gene Research, Hubei Hongshan Laboratory, Huazhong Agricultural University, Wuhan, Hubei 430070, China.

[2]State Key Laboratory of Biocatalysis and Enzyme Engineering, Hubei Collaborative Innovation Center for Green Transformation of Bio-Resources, Hubei Key Laboratory of Industrial Biotechnology, School of Life Sciences, Hubei University, Wuhan, Hubei 430062, China.

*Correspondence:

Ping Yin (yinping@mail.hzau.edu.cn) or Shan Wu (wushan91@hubu.edu.cn)

[3]These authors contributed equally.

This file includes:

Supplementary Figures. S1 to S10

Supplementary Table S1

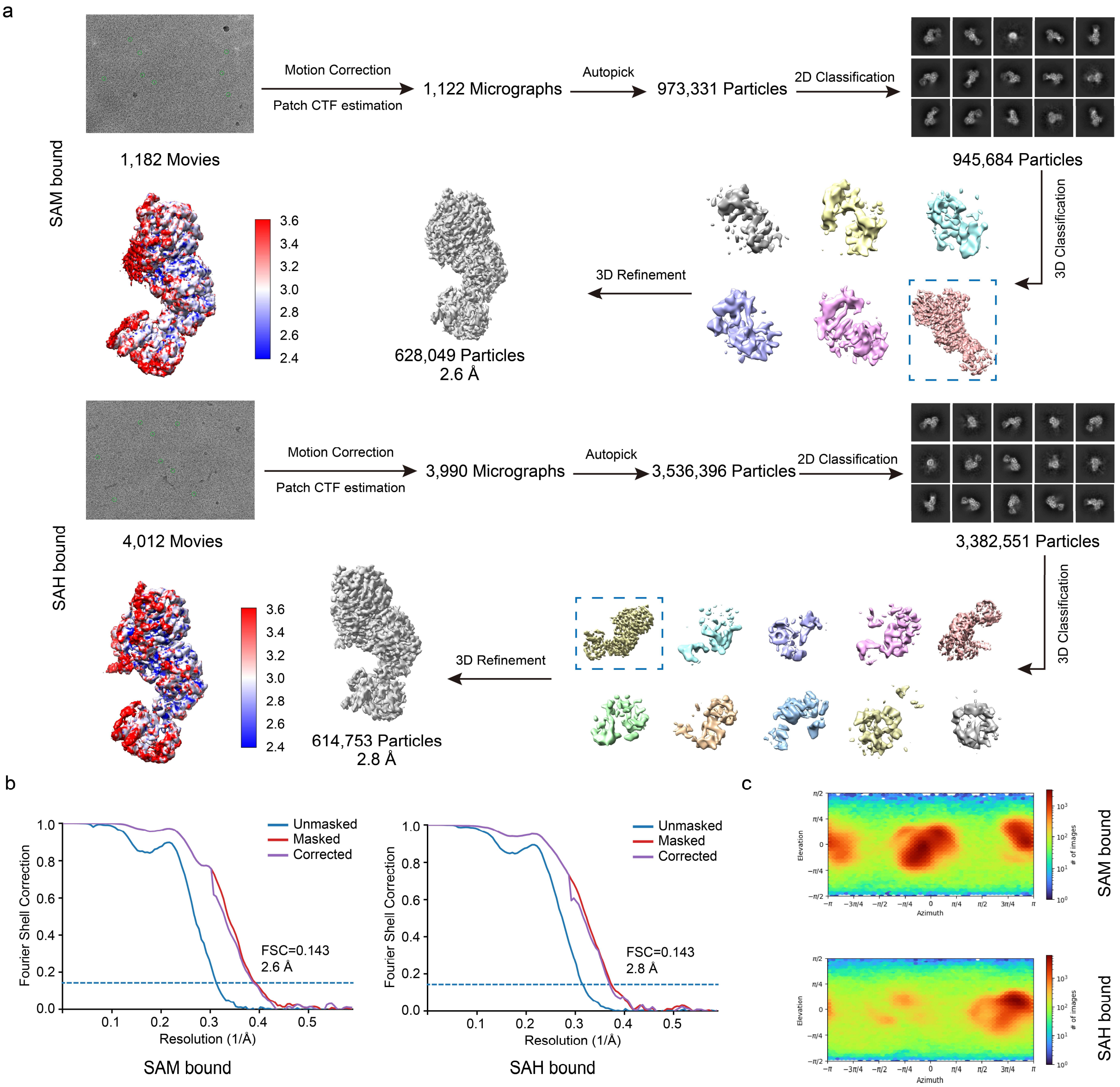

Supplementary Fig. S1 Cryo-EM data processing of the SAM and SAH-bound MTA1 complex. a Flowchart for cryo-EM data processing of the SAM and SAH-bound MTA1 complex. See Methods for detailed information. Local resolution of the Cryo-EM maps generated by ResMap colored from blue to red to indicate resolution from high to low. The highest resolution of the EM maps of SAM and SAH-bound complexes reach 2.6 Å and 2.80 Å, respectively. **b** The Gold-standard Fourier Shell Correlation curves of the final refined models of the SAM and SAH-bound MTA1 complex. **c** Angular distribution for the final reconstruction of the SAM and SAH-bound MTA1 complex.

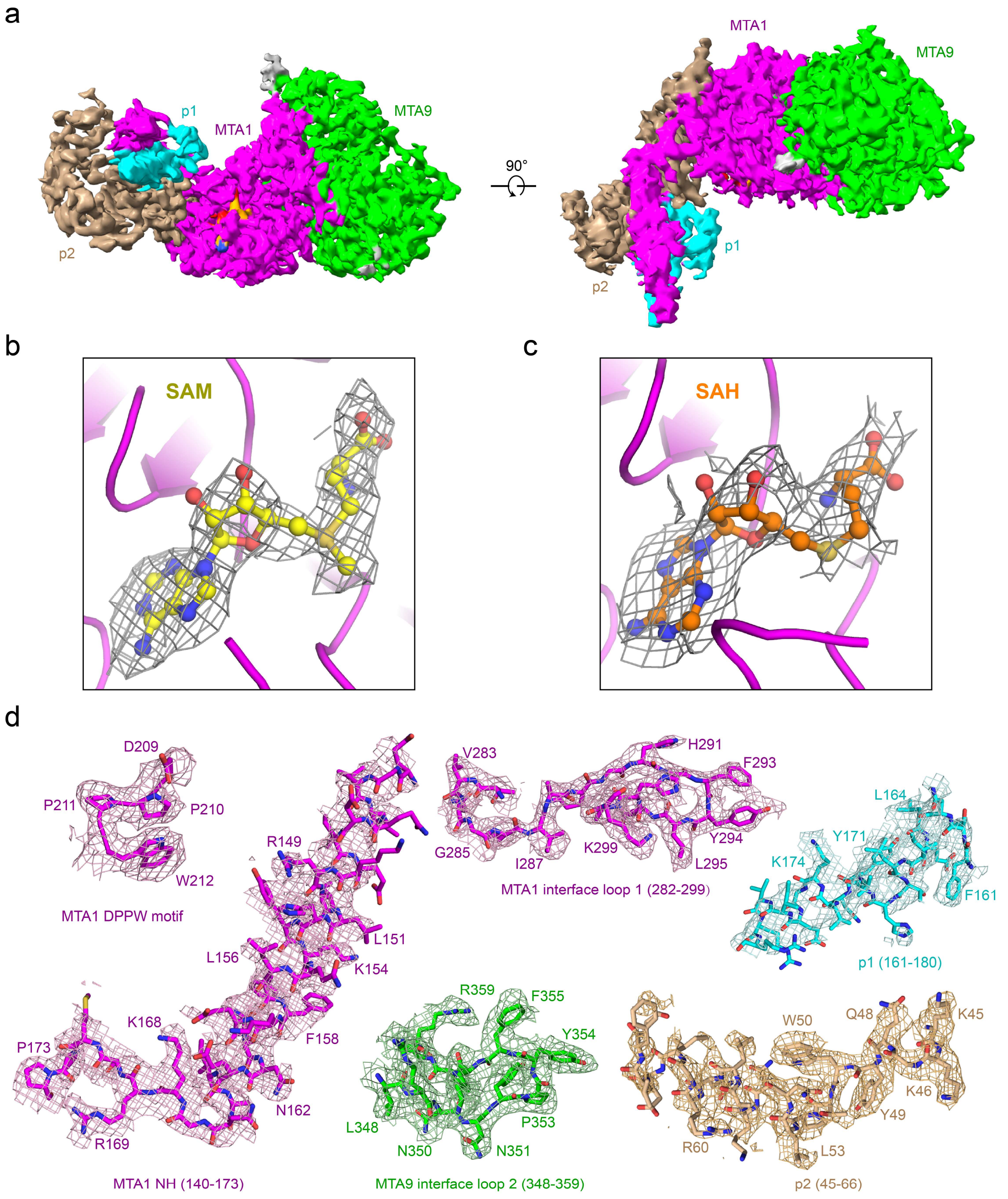

Supplementary Fig. S2 Cryo-EM map of the MTA1 complex. a Views of the cryo-EM maps of the SAH-bound MTA1 complex. **b** Cryo-EM density for SAM in the SAM-bound MTA1 complex. **c** Cryo-EM density for SAH in the SAH-bound MTA1 complex. **d** Representative Cryo-EM density for key components of the MTA1 complex. NH, N-terminal helix.

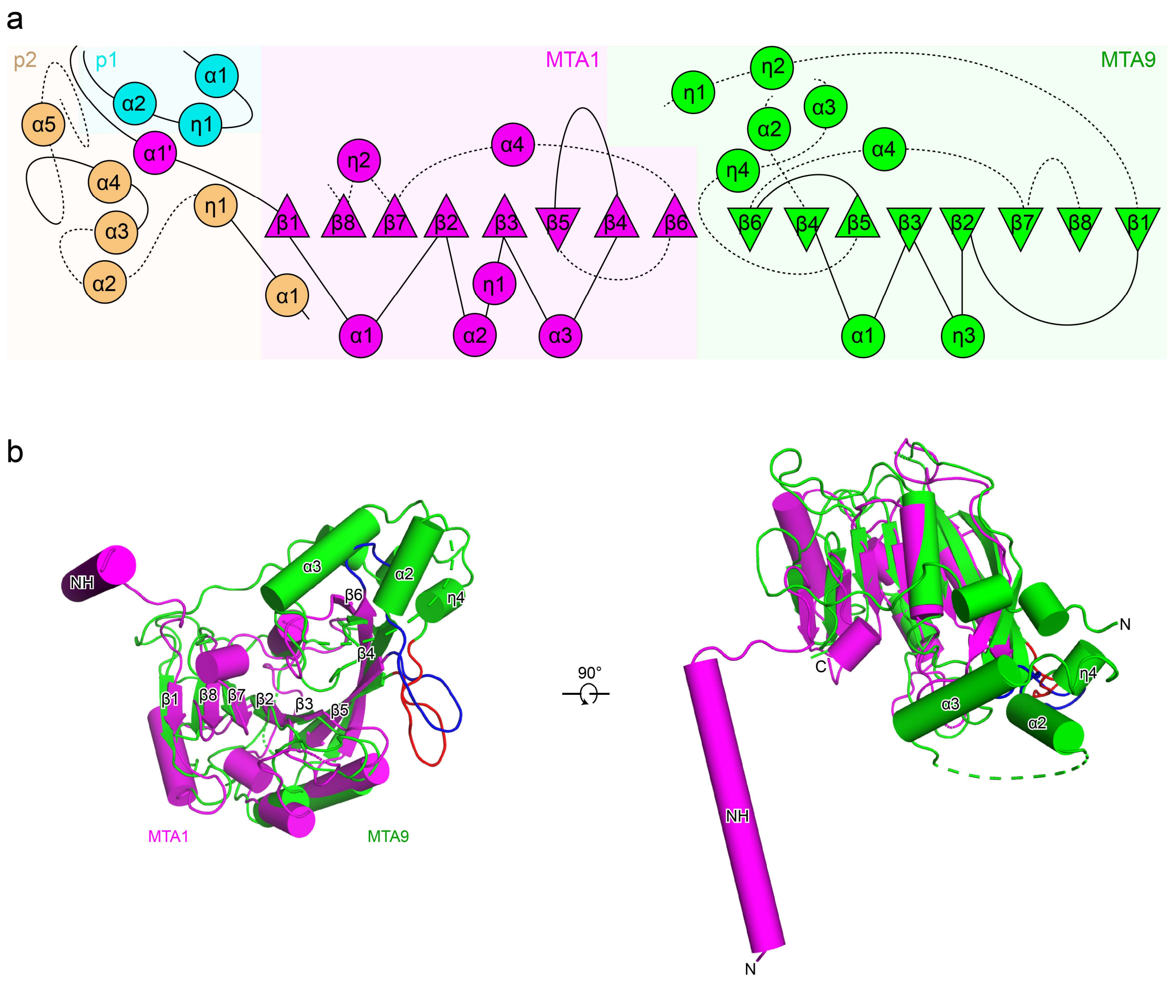

Supplementary Fig. S3 Structure analysis of the MTA1 complex. a Diagram of the MTA1 complex secondary structure profiles. MTA1 (magenta), MTA9 (green), p1 (cyan) and p2 (wheat) are boxed with light colored backgrounds. The MTase domain of MTA1 contains an eight-stranded β-sheet (triangles) flanked by four α-helices and two 3_{10}-helices (circles). Structural elements are numbered by their linear order in the sequence. The loops in the front are indicated by black lines, and loops in the back are indicated by black dashed lines. **b** Structural comparison of MTA1 (magenta) and MTA9 (green). Two perpendicular views of superimposed MTA1 and MTA9 colored magenta and green, respectively. The main differences between the MTase domains of MTA1 and MTA9 are the less conserved regions (α2, α3, η4 in MTA9) between β4 and β5.

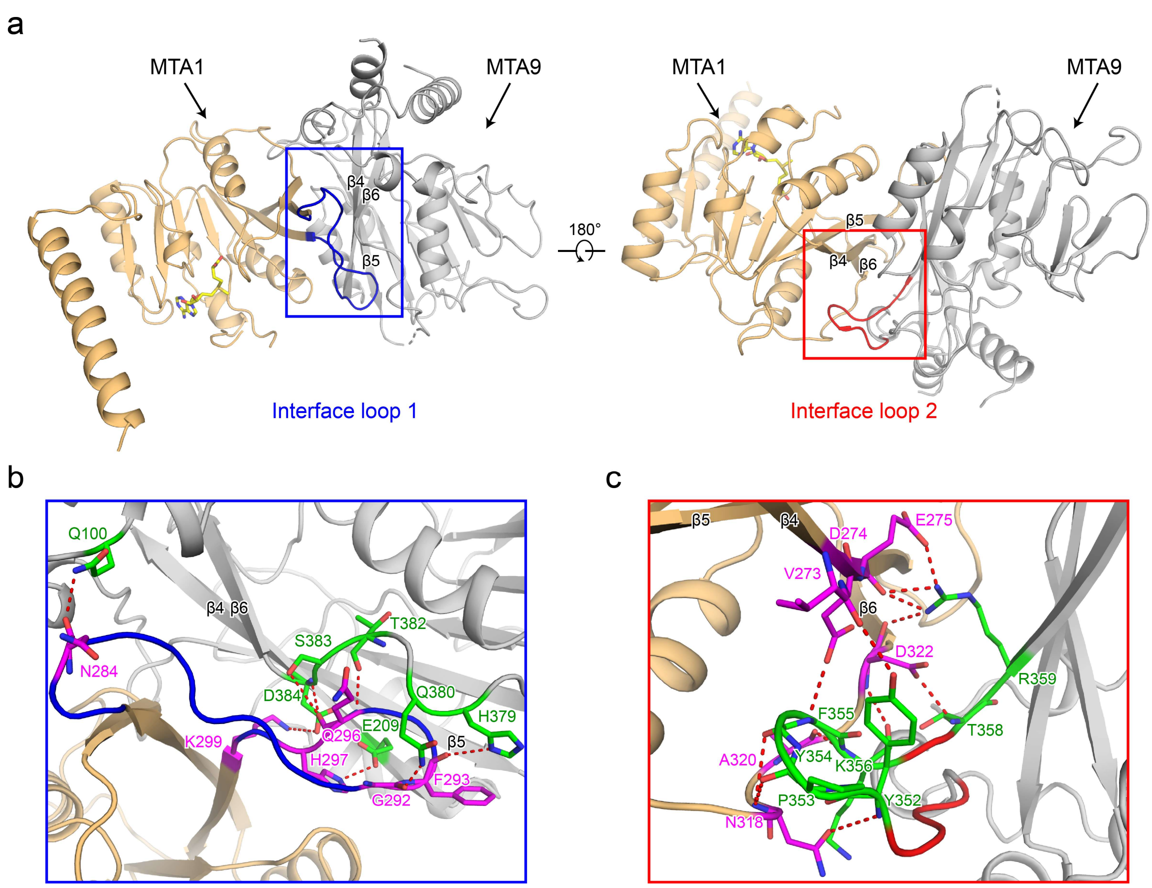

Supplementary Fig. S4 Extensive hydrogen network between MTA1 and MTA9 in the MTA1 complex. a Interface loop 1 (boxed with a blue rectangle) and interface loop 2 (boxed with a red rectangle) that mediate the interactions between MTA1 and MTA9. MTA1 and MTA9 are coloured wheat and silver, respectively. **b, c** Details of interactions mediated by interfaces loop 1 (blue) and 2 (red). Hydrogen bonds are represented by red dashed lines. Residues from MTA1 (magenta) and MTA9 (green) that are involved in interactions are shown as sticks.

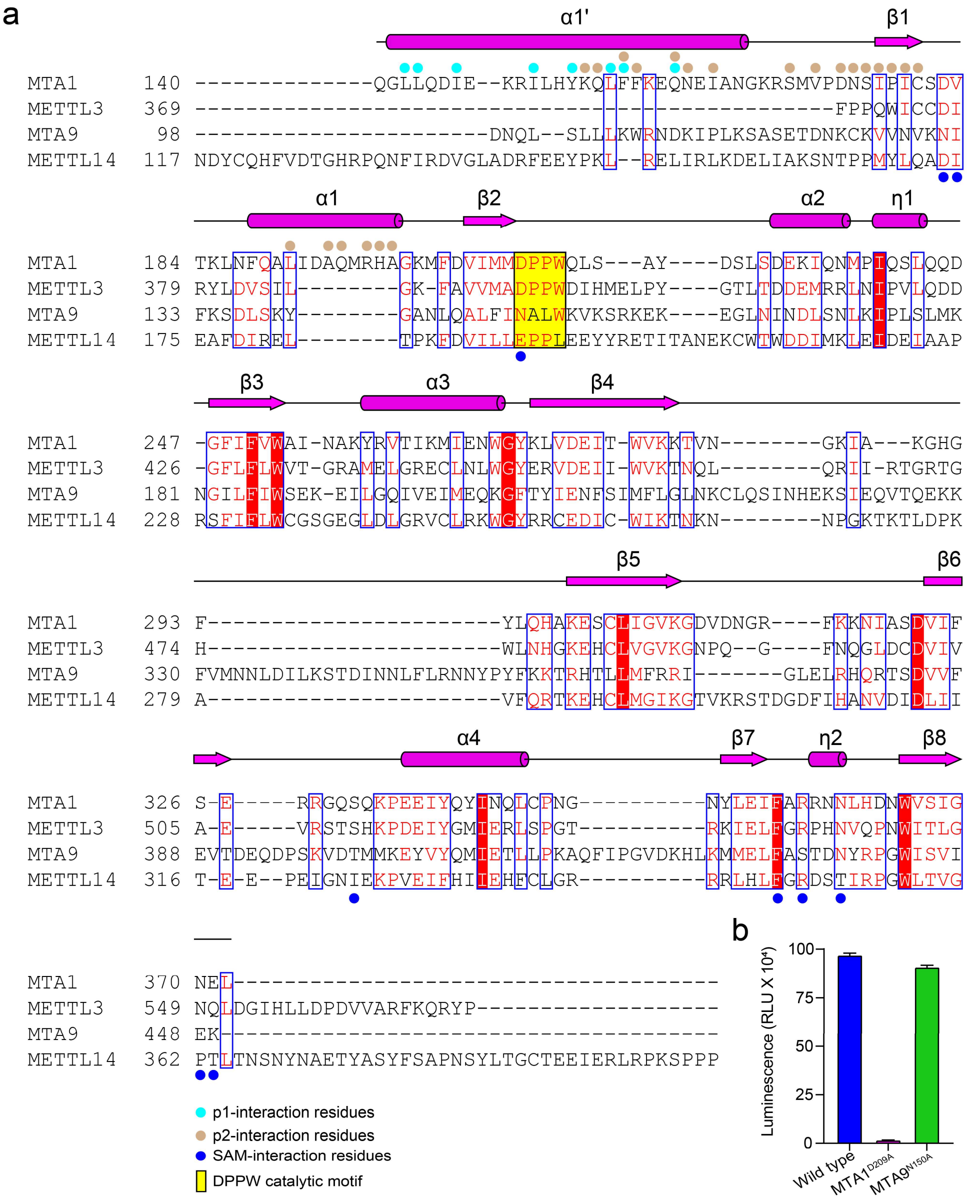

Supplementary Fig. S5 Sequence similarity and mutagenesis analysis of the MT-A70 proteins. a Sequence alignment of *T. thermophila* MTA1 and MTA9, Homo sapiens METTL3 and METTL14. The alignment was generated using the MUSCLE and ENDscript programs. Secondary structural elements of MTA1 are shown on top. Sequence identity is shown in white letters with a red background, and sequence similarity is shown in red letters. Cyan and wheat dots above represent residues of MTA1 that are involved in interaction with p1 and p2, respectively. Blue dots below indicate residues of MTA1 that interact with SAM. The DPPW catalytic motif is shown in letters with yellow background. **b** Effects of D209A (in the DPPW motif) of MTA1 and N150A (in the eroded NALW motif) of MTA9 in the MTA1 complex on

DNA methylation. The error bars indicate the SEM of three independent measurements. This experiment was repeated twice.

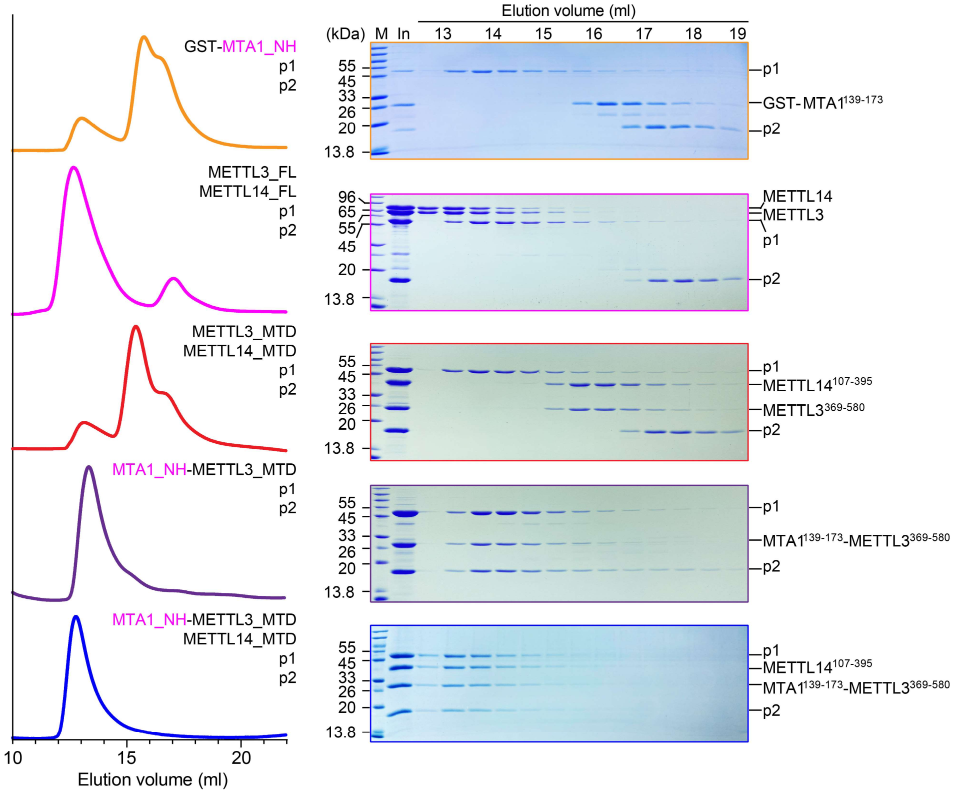

Supplementary Fig. S6 SEC analysis for the NH fusing assay. All the runs were performed in a Superdex-200 Increase 10/300 GL column. Proteins in each run were mixed at an equimolar ratio and subjected to SEC analysis. Left panel shows the SEC of the protein mixture. Coomassie blue-stained SDS-PAGE gels of peak fractions are on the right of corresponding SEC. NH, N-terminal helix; MTD MTase domain; FL, full-length.

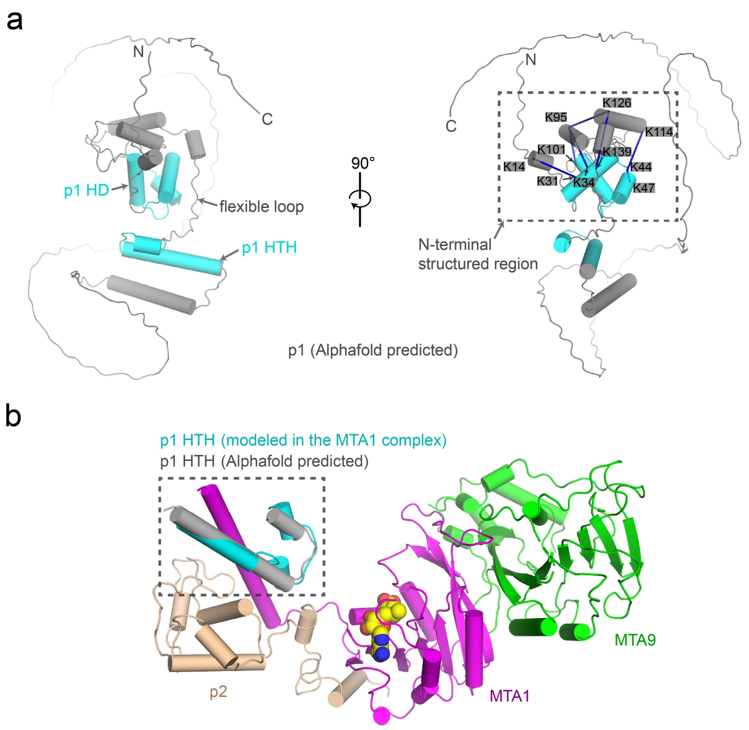

Supplementary Fig. S7 Analysis of the p1 Alphafold structure. a Two views of the Alphafold structure of p1. Left: The HD (cyan; residues 25-71) and HTH (cyan; residues 152-183) of p1 are connected by a flexible loop (gray). Right: Cross-links mapped on the Alphafold structure of p1 are consistent with a cutoff of 26 Å, which indicates a structured region (including the HD) at the N-terminus of p1. HD, homeobox-like domain; HTH, helix-turn-helix-like motif. **b** Structural comparison of the Alphafold predicted p1 HTH (gray) and cryo-EM determined p1 HTH (cyan) in the MTA1 complex.

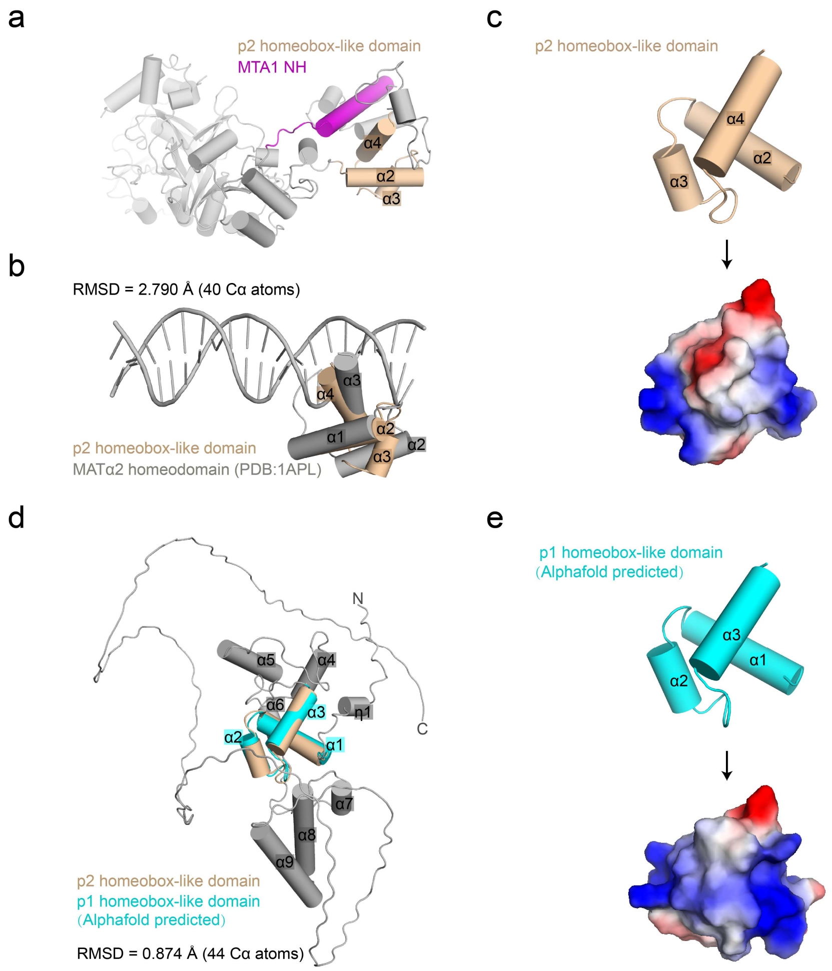

Supplementary Fig. S8 Analysis of the homeobox-like domain of p1 and p2. a Homeobox-like domain of p2 (wheat; residues 50-96) in the MTA1 complex. The NH of MTA1 is shown in magenta. **b** Superposition of the p2 homeobox-like domain (wheat) and the MATα2 homeodomain (gray; PDB:1APL). **c** The surface electrostatic potential of the p2 homeobox-like domain. **d** Superposition of the Alphafold predicted p1 homeobox-like domain (cyan; including α1, α2 and α3) and cryo-EM determined p2 homeobox-like domain (wheat). **e** The surface electrostatic potential of the p1 homeobox-like domain.

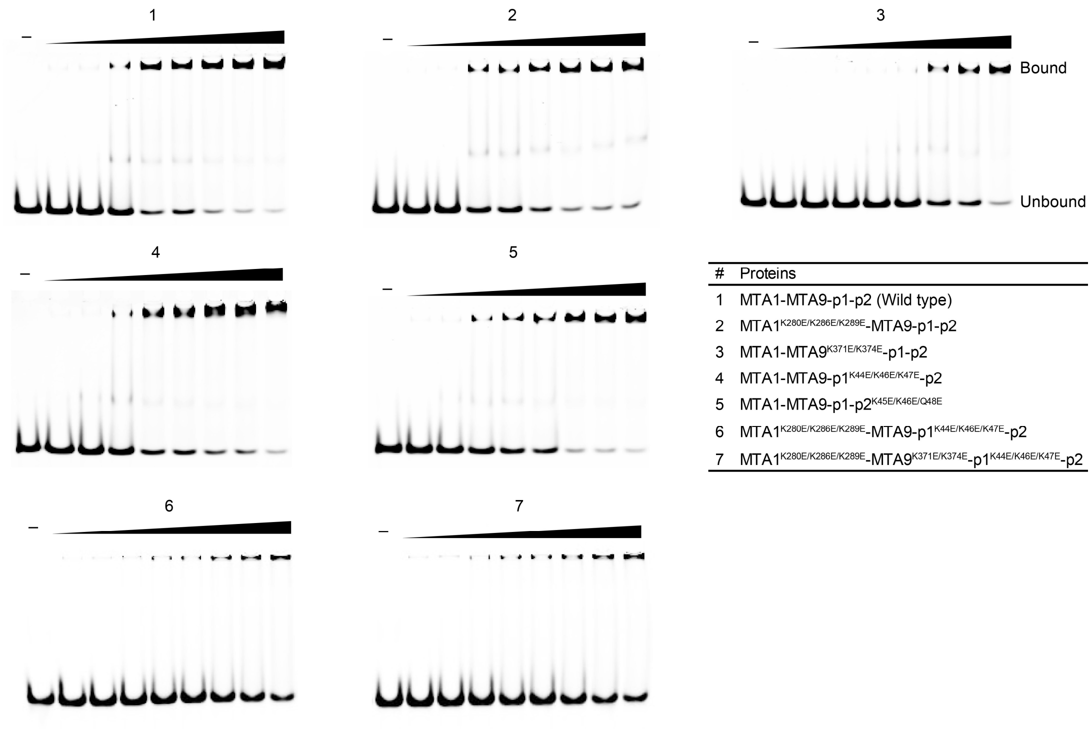

#	Proteins
1	MTA1-MTA9-p1-p2 (Wild type)
2	$MTA1^{K280E/K286E/K289E}$-MTA9-p1-p2
3	MTA1-$MTA9^{K371E/K374E}$-p1-p2
4	MTA1-MTA9-$p1^{K44E/K46E/K47E}$-p2
5	MTA1-MTA9-p1-$p2^{K45E/K46E/Q48E}$
6	$MTA1^{K280E/K286E/K289E}$-MTA9-$p1^{K44E/K46E/K47E}$-p2
7	$MTA1^{K280E/K286E/K289E}$-$MTA9^{K371E/K374E}$-$p1^{K44E/K46E/K47E}$-p2

Supplementary Fig. S9 EMSA analysis of the interaction between DNA and the MTA1 complex containing indicated MTA1, MTA9, p1 or p2 mutations. The DNA was labeled by 6-Carboxyfluorescein (FAM) at the 5′ end. 500 nM dsDNA was incubated with increasing amounts of the WT or mutants.

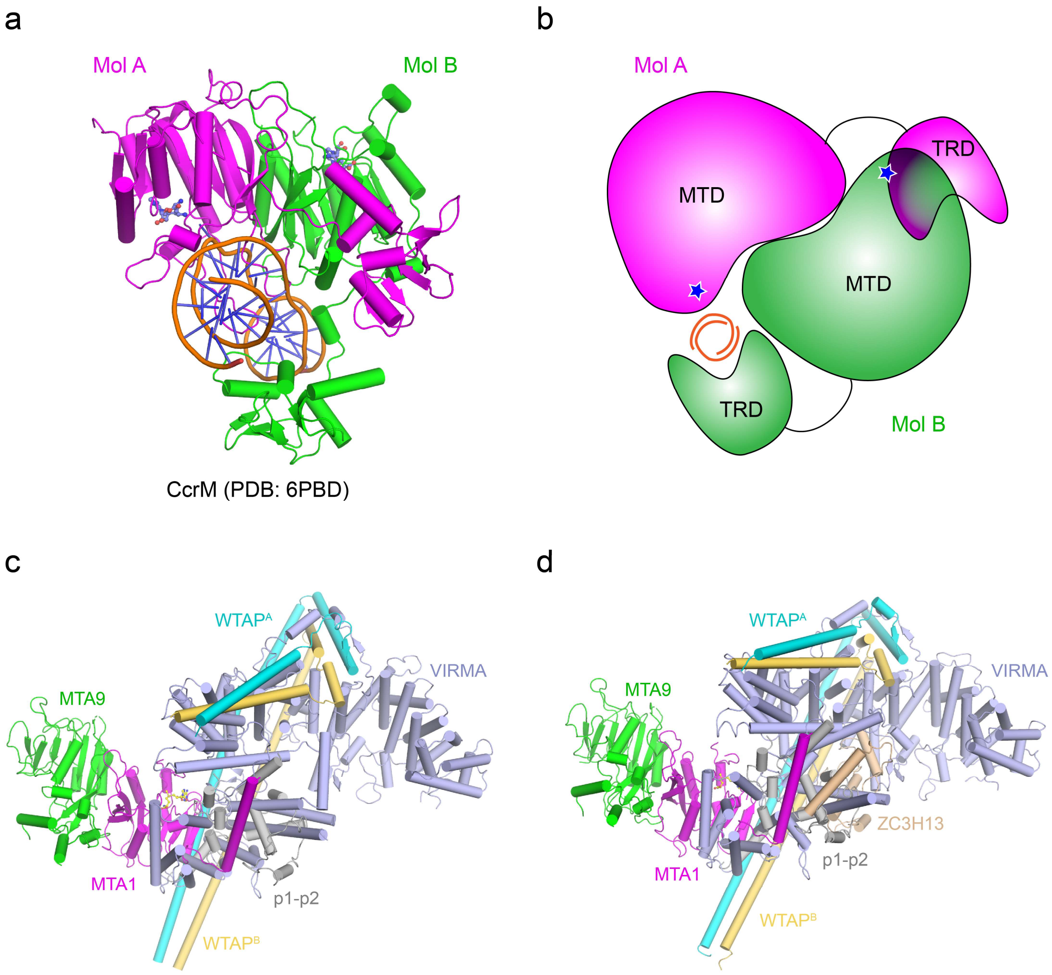

Supplementary Fig. S10 Comparison of MTA1 complex and other MTases. a Dimeric structure of class-β 6mA MTase CcrM. CcrM-DNA complex. Molecule A and Molecule B of the dimeric CcrM are shown in magenta and green, respectively. The sugar-phosphate backbone of DNA substrate is shown in orange. The DNA bases and co-factor sinefungin (a SAM analog) are shown in blue. **b** A working model of the dimeric CcrM. The dimeric CcrM use a "division of labor" between its two molecules. The MTD from Molecule A (magenta) and the TRD from the Molecule B (green) are face to face and establish a functional surface for DNA recognition and methylation. The DNA substrate is shown as orange ribbon. Sinefungin is indicated as a blue star. MTD, MTase domain; TRD, target recognition domain. **c, d** Superposition of p1-p2 in the MTA1 complex and m^{6}A writer complex regulatory complexes including (**c**) WTAP-VIRMA (PDB: 7VF5, 7YG4) or (**d**) WTAP-VIRMA-ZC3H13 (PDB: 7VF2). MTA1, magenta; MTA9, green; p1 and p2, gray; WTAPA, cyan; WTAPB, yellow; VIRMA, light blue; ZC3H13, wheat.

Table S1. Cryo-EM data collection and refinement statistics

	RT<br>PDB: 7YI9<br>EMBD: EMD-33854	RT<br>PDB: 7YI8<br>EMBD: EMD-33853
**Data collection and processing**		
Microscope	Krios	Krios
Voltage (kV)	300	300
Camera	Gatan K3	Gatan K3
Magnification	105,000	105,000
Pixel size at detector (Å/pixel)	0.85	0.85
Total electron exposure (e^-/Å^2)	55.1	55.1
Frames collected during exposure (no.)	40	40
Defocus range (µm)	-1.0~-1.5	-1.0~-1.5
Automation software	EPU	EPU
Micrographs collected (no.)	1,182	4,012
Micrographs used (no.)	1,122	3,990
Total extract particles (no.)	973,331	3,536,396
**For each reconstruction**		
Refined particles (no.)	628,049	614,753
Final particles (no.)	628,049	614,753
Point group	C1	C1
Resolution (global, Å)		
FSC 0.5 (unmasked/masked)	3.8/3.4	3.7/3.3
FSC 0.143 (unmasked/masked)	3.3/2.6	3.2/2.7
Resolution range (local, Å)		
Map sharpening B factor (Å^2)	113.5	106.8
Map sharpening methods	Half-maps correlation	Half-maps correlation
**Model composition**		
Protein	634	634
Ligands	1	1
**Model refinement**		
Refinement package	PHENIX	PHENIX
- real or reciprocal space	Real Space	Real Space
- resolution cutoff	2.6	2.7
Model-Map scores		
- CC	0.75	0.74
B factors (Å^2)		

Protein residues	71.36	43.04
Ligands/DNA/RNA	58.47	30.75
R.m.s. deviations from ideal values		
Bonds length (Å)	0.003	0.002
Bond angles (°)	0.606	0.553
**Validation**		
MolProbity score	1.85	1.79
CaBLAM outliers	2.64	2.3
Clashscore	9.63	9.73
Poor rotamers (%)	0	0
C-beta deviations	0	0
EMRinger score (if better than 4 Å resolution)		
Ramachandran Plot		
Favored (%)	95.00	95.97
Outliers (%)	0	0
